# The Energy Expenditure of Sedentary Behavior: A Whole Room Calorimeter Study

**DOI:** 10.1371/journal.pone.0063171

**Published:** 2013-05-03

**Authors:** Robert L. Newton, Hongmei Han, Theodore Zderic, Marc Hamilton

**Affiliations:** Preventive Medicine and Healthy Aging, Pennington Biomedical Research Center, Baton Rouge, Louisiana, United States of America; University of Sao Paulo, Brazil

## Abstract

It has recently been recommended that sedentary behavior be defined as sitting or reclining activities expending less than 1.5 metabolic equivalents (METs), which is distinct from the traditional viewpoint based on insufficient moderate-vigorous activity or formal exercise. This study was designed to determine the energy expenditure associated with common sedentary behaviors. Twenty-five African American adults (BMI 27.8±5.5) participated in the metabolic chamber study. Participants entered the metabolic chamber in the morning and their basal metabolic rate was estimated. They were fed breakfast and then engaged in four different sedentary behaviors sequentially, lasting 30 minutes each. The activities included reclining, watching TV, reading, and typing on a computer. In the afternoon, the participants were fed lunch and then the activities were repeated. The results show that the energy expenditure values between the morning and afternoon sessions were not significantly different (p = .232). The mean energy expenditure of postprandial reclining (0.97 METs) was slightly, but significantly, lower than postprandial watching TV (p = .021) and typing (p<.001). There were no differences in energy cost (1.03–1.06 METs) between the seated (i.e., reading, typing, watching TV) sedentary activities. The energy expenditure of several common sedentary behaviors was approximately 1.0 METs in the postprandial state. The results support the conclusion that the average energy cost of common sedentary behaviors is narrowly banded around 1.0 METs in the postprandial state.

## Introduction

There has been a recent emergence of studies assessing the health consequences of sedentary behavior (sitting), as distinct from too little exercise (sustained moderate-vigorous aerobic activity), and the physiology underlying these associations. [1]–[7] Evidence has been consistent in showing that large amounts of sedentary behavior are positively associated with the risk of coronary heart disease, [8] hypertension, [9] diabetes, [10] metabolic syndrome, [11], [12] obesity, [13], [14], mortality, [15], [16] and some cancers. [16], [17] From this background, insights about the seemingly simple issue regarding sitting energy expenditure are remarkably fundamental to many researchers. The plausibility that the observational associations are actually caused directly by sedentary behavior is strengthened by mounting physiological and molecular insights from experimental studies.[7], [18]–[22].

These epidemiological and experimental findings have led researchers to define “sedentary” largely in terms of posture and concurrent energy expenditure as a helpful starting point. The issue has become so pressing that there was a recent consensus group recommending that journals require authors to define “sedentary” as sitting or reclining activity ≤1.5 metabolic equivalents (METs). [23] Indeed, it is very well established that basal metabolic rate (BMR) is typically 0.7–1.0 METs, depending in large part on body composition. However, it is ironic that despite sitting being a ubiquitous behavior in all people, there are relatively few well-controlled studies actually assessing the energy expenditure of sitting.[24]–[32] This is out of balance with the robust physical activity literature describing exercise and other more demanding forms of lifestyle activity. [33], [34] However, sitting (as one watches TV, reads, and types on a computer) alone may account for more than 9–10 hours of many individuals’ waking days, and thus accurate assessment of the metabolic rate during sitting is essential in order to minimize errors in estimates of sedentary behavior [35].

Most of the studies that have assessed the energy expenditure of sitting behaviors[24]–[32] were focused on comparing basal metabolic rates to the energy cost of light or moderate intensity physical activity and included one or two sedentary behaviors. TV viewing has been frequently assessed in these studies, whereas other common sedentary activities, such as typing and reading, are less well studied. These studies also assessed sedentary behaviors over short periods of time, limiting their ability to detect spontaneous “fidgeting” and other simple behaviors requiring energy beyond sitting, which have been shown to increase energy expenditure. [36] In addition, metabolic carts that require the participant to be fixed to a hose and mouthpiece/facemask have typically been utilized to measure energy expenditure as opposed to less restrictive whole room calorimeters. Furthermore, most measured each activity only once, measurements were made exclusively in the fasted state, and most participants have been of European decent. These issues suggest that there are several important considerations for studies attempting to provide accurate estimates of sitting energy expenditure. First, quantifying sitting energy expenditure should be done at multiple times in the day (e.g. the morning and afternoon) because the percentage of time spent sitting is greatest during the workday in the modern chair-dominated office environments. [37] Second, since modern populations spend most of their time in the postprandial state (i.e., for >5 hours after meals), it is best to conduct estimates of sitting energy expenditure in the postprandial state. Third, investigators should use whole room metabolic chambers to estimate energy expenditure, whenever possible. This method allows researchers to simulate real-world activities in a more naturalistic setting. Fourth, researchers should assess sitting energy expenditure in at-risk populations in order to provide generalizability of the findings. Therefore, the present study will assess sitting energy expenditure in the morning and afternoon, in the postprandial state, and will utilize the metabolic chamber in a sample of African American participants. In these ways, the current study of sitting energy expenditure can play a significant role in advancing the field of inactivity physiology.

The present study was designed to determine the energy expenditure of common sedentary behaviors. All of the activities were conducted in a metabolic chamber over an 8 hour period. Our methodology involved a protocol where each person reclined/sat for extended periods of time while performing common sedentary activities. The intent was to determine the average energy expenditure of these sitting behaviors.

## Methods

### Ethics Statement

Each volunteer provided written informed consent and all procedures were approved by the Pennington Biomedical Research Center Institutional Review Board.

### Participants

In order to be included into the study, participants had to a) self-classify themselves as African American; b) be 18 years and older; c) be free of serious medical conditions; d) be free of any condition that would prevent them from engaging in physical activity, and e) weigh less than 250 lbs. Participants were primarily recruited through email and online advertisements, as well as through presentations at community events and newspaper advertisements.

### Measures

#### Body Mass Index (BMI)

Height and weight of each participant was measured in normal clothing, without shoes and socks. Standing height was measured to the nearest 0.1 centimeter (cm) by a wall-mounted stadiometer (Holtain Ltd., Crymych, Dyfed, UK). Weight was measured to the nearest 0.1 kilogram (kg) using an Indiana Scale Company model GSE 450 digital scale. Body mass index (BMI) was calculated by dividing the participant’s weight in kilograms by the square of their height in meters (kg/m^2^).

#### Metabolic chamber

There were two metabolic chambers on site measuring 10′x14′x8′ (27,000 L). [38] Once a month, the accuracy and precision of the respiratory chambers are assessed by 24-h propane combustion tests. The chamber software allows for the measurement of energy expenditure with high time resolution [38] by detecting changes in energy expenditure due to changes in activity level. The chamber responds to changes from one steady state of respiration to another and correctly averages repeated changes in respiration with periods less than 15 minutes (<1.4% error for alternating O_2_ consumption levels).

Collected data was stored on a computer and the last 20 minutes of each 30 minute time block was averaged across the two repetitions (mL O_2_/min, kcal/min), normalized to body mass (mL O_2_/kg/min, kcal/kg/hr). Excluding the first 10 minutes was necessary to ensure complete equilibration of energy expenditure traces between activity levels.

### Procedures

Participants entered the chamber at 8:00 a.m. after a 12-hour fast (they were instructed not to consume any food or liquids, except water, after 8:00 pm the previous evening). Baseline resting energy expenditure was calculated while participants reclined on a bed for 30 minutes in the thermo neutral environment of the chamber. Participants were allowed 30 minutes to consume a 400 kcal breakfast (16% protein, 55% carbohydrate, 28% fat), which was served immediately after the baseline period. The participants then engaged in the following activities for 30 minutes at a time: reclining/resting, watching TV, sitting at a desk, reading a book (sedentary activities), walking on a treadmill at a pace of 1.5 miles per hour (light intensity physical activity), and walking at 3 miles per hour on the treadmill (moderate intensity physical activity). Participants were then allowed 30 minutes to consume a 624 kcal lunch (12% protein, 45% carbohydrate, 43% fat) and each activity was repeated in the afternoon. Participants left the chamber at approximately 4:30 p.m. after completing all activities. The exact schedule for the metabolic chamber stay is displayed in Table 1. Although the treadmill walking was a part of the overall study design and ensured the subjects did have a modest amount of physical activity during the testing period, these results are extraneous to the purpose of the present investigation and will be presented elsewhere.

**Table 1:** Schedule of activity during the metabolic chamber stay.

8:00 AM	Enter chamber	
8:30 AM	Energy expenditure data collection begins	Quietly resting on bed (preprandial reclining)
9:00 AM	Breakfast	
9:30 AM	Replicate 1	Quietly resting on bed (postprandial reclining)
10:00 AM		Watching TV
10:30 AM		Typing at a desk
11:00 AM		Reading at a desk
11:30 AM		Treadmill walking 1.5 mi/hr^a^
12:00 PM		Treadmill walking 3 mi/hr^a^
12:30 PM	Lunch	
1:00 PM	Replicate 2	Quietly resting on the bed (postprandial reclining)
1:30 PM		Watching TV
2:00 PM		Typing at a desk
2:30 PM		Reading at a desk
3:00 PM		Treadmill walking 1.5 mi/hr^a^
3:30 PM		Treadmill walking 3 mi/hr^a^
4:00 PM		Quietly resting on bed a
4:30 PM	Energy expenditure collection ends	

a These data were not included in the current investigation.

### Statistical Analysis

Intraclass correlation coefficients (ICC) for each sedentary behavior were calculated for the morning and afternoon sessions. Mixed models ANOVA was used to compare energy expenditure amongst each of the sedentary behaviors between the morning and afternoon sessions. Mixed models ANOVA was also used to compare energy expenditure between the estimated basal metabolic rate and each sedentary activity utilizing Tukey-Kramer post-hoc adjustments. All statistical analyses were performed using SAS 9.2.

## Results

### Demographics

There were 25 participants (40% male) who were recruited for the study. All participants completed the study (Table 2). The participants had an average BMI of 27.8 (5.5).

**Table 2:** Characteristics of participants (n = 25).

Variable	Mean	SD	Minimum	Maximum
Age	38.2	11.4	20.0	56.0
Height (cm)	168.5	7.2	155.1	182.2
Weight (kg)	79.0	16.7	56.3	113.3
BMI	27.8	5.5	19.9	41.1

### Stability of Sedentary Activities

The ICCs for each sedentary behavior were 0.77, 0.77, 0.81 and 0.70 for reclining, watching TV, typing and reading, respectively, when measured as kcal/kg/hr. The values were essentially equal when measured as mL O_2_/kg/min.

### Comparison of Morning and Afternoon Sessions

When assessed as kcal/kg/hr, the overall effect for activity type (F(3, 59.6) = 5.79; p = 0.002) was significant, but the effects of repetition (F(1, 25) = 1.50; p = 0.232), and the interaction of activity type by repetition (F(3, 90.2) = 2.36; p = 0.077) were not. The overall activity type effect indicates that there were differences in energy expenditure across the sedentary activities, and the interaction trend suggests that these may have differed across the morning and afternoon. The lack of a repetition effect indicates that energy expenditure values between the morning and afternoon sessions were not significantly different. These same effects were seen when the data was expressed as mL O_2_/kg/min. Therefore, the average value of the morning and afternoon sessions were utilized in subsequent analyses.

### Comparison of Sedentary Behaviors

When assessed as kcal/kg/hr, there was a significant effect for activity type (F(4, 96) = 47.3; p<0.001). Post-hoc tests with Tukey-Kramer adjustments showed that the energy expenditure of preprandial reclining (estimated BMR) was significantly lower than each of the postprandial sedentary behaviors (all p values ≤0.001). In addition, the kcal/kg/hr of postprandial reclining was significantly lower than watching TV (p = 0.021) and typing (p<0.001). There were no differences between the seated (i.e. reading, typing, watching TV) sedentary behaviors. These results were identical when expressed as mL O_2_/kg/min and METs (Table 3).

**Table 3:** Energy expenditure of sedentary activities.

Activity	N	mL O_2_/kg/minMean±SE	Kcal/kg/hrMean±SE	METsMean±SE
Preprandial				
Reclining	24	2.88±0.09^a^	0.86±0.03^a^	0.82±0.03^a^
Postprandial				
Reclining	24	3.41±0.10^b^	1.01±0.03^b^	0.97±0.03^b^
Watching TV	24	3.61±0.12^c^	1.08±0.04^c^	1.03±0.03^c^
Typing	24	3.70±0.12^c^	1.11±0.04^c^	1.06±0.03^c^
Reading	24	3.64±0.14^bc^	1.08±0.04^bc^	1.04±0.04^bc^

Note: Values with different superscripts are significantly different from one another.

**Figure 1:**
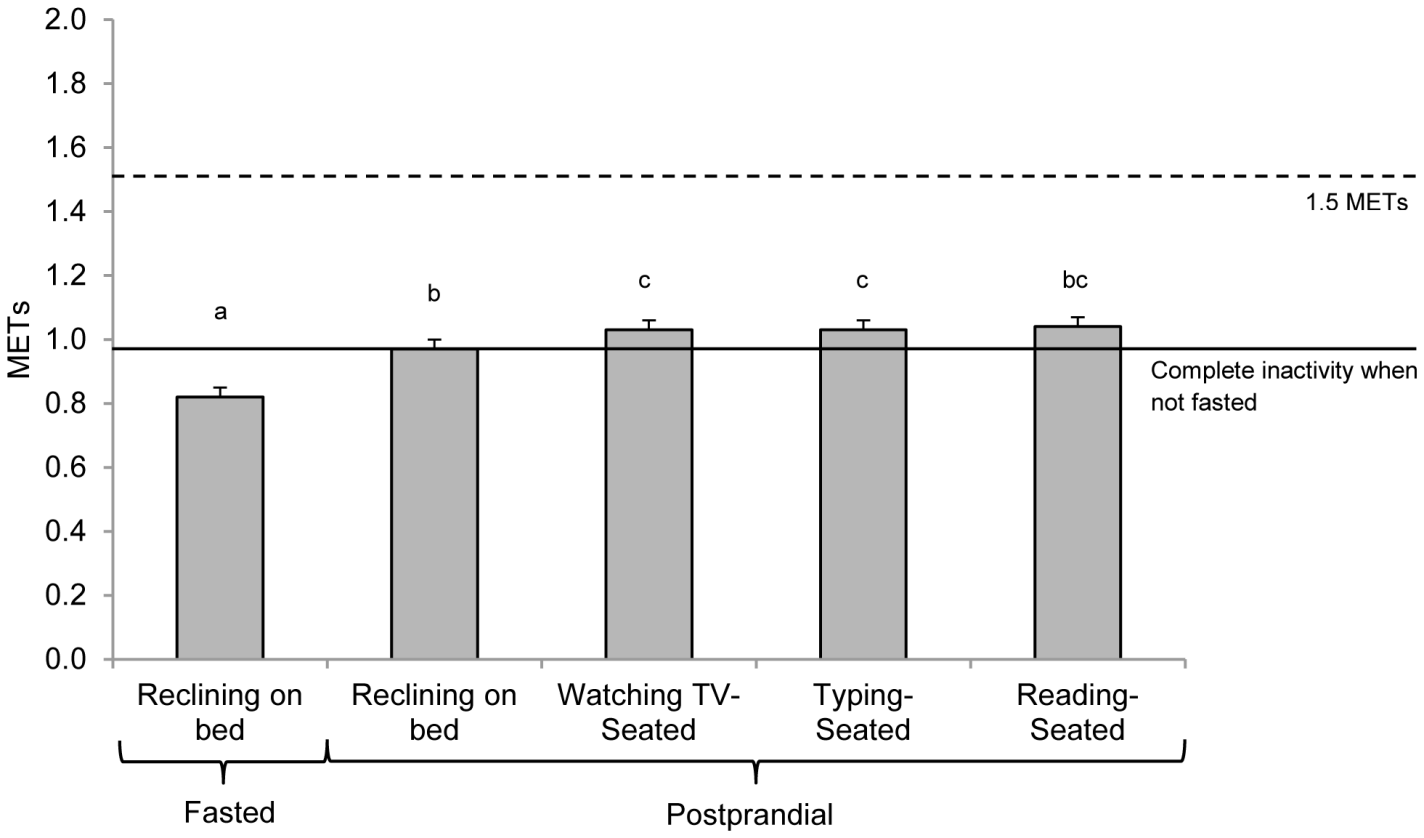
Oxygen consumption in MET units during sedentary behaviors. Each activity was measured for 30 minutes following breakfast and lunch. Bars represent the average MET value for each sedentary activity across breakfast and lunch. The dashed line represents the recommended MET value for sedentary behaviors. The solid line represents the average MET value for postprandial reclining. One MET is by definition is 3.5 ml O_2_/kg/min. Postprandial seated activities on average required a 7% greater metabolic rate than postprandial reclining, and the average of the four sedentary behaviors in the postprandial state was 1.02 METs. Note: Values with different superscripts are significantly different from one another.

The increase in oxygen consumption and energy expenditure from pre- to postprandial reclining was ∼18% (p<0.001). To determine the effect of the sitting activities on energy expenditure independent of the feeding state we compared the postprandial reclining conditions to the postprandial seated conditions. The oxygen consumption and energy expenditure of common seated activities was on average 7% (0.07 kcal/kg/hr) greater than the reclining posture (p<0.001). In Figure 1, the average METs (mL O_2_/kg/min/3.5) of each seated activity are reported in relation to the recommended 1.5 METs threshold for defining sedentary activity. [23].

## Discussion

The data from the current study shows that the energy expenditure of several common sedentary behaviors when reclining or sitting ranges from 1.01 to 1.11 kcal/kg (body weight)/hr in the postprandial state. Our study is not the first to assess the energy expenditure of sitting behaviors.[24]–[32] These other studies have shown that the energy cost of sitting ranges from 0.99 to 1.14 kcal/kg/hr. [25], [26], [28], [39], [40] Multiple studies have specifically assessed the energy cost of TV viewing, and obtained values of 0.93 kcal/kg/hr [28], 0.97 kcal/kg/hr, [39], 1.03 kcal/kg/hr (the energy cost of playing a sedentary video game) [41], and 1.08 kcal/kg/hr. [40] which are similar, but on average slightly below our value of 1.08. These findings show that the data from the current study is consistent with that of previous investigations. However, there are several important issues that the current study highlights that should be considered when interpreting the results of energy expenditure studies, including the stability of the energy expenditure estimates, the thermic effect of food, and the study sample.

The findings from the current study indicate that energy expenditure of sedentary behaviors is stable across the day, even when accounting for the postprandial rise. This was partially demonstrated by the moderate to strong ICCs which indicated that very little variability was due to the time of day. These correlations may have been slightly stronger if the energy cost of the meals was identical. This conclusion is also supported by the ANOVA data which demonstrated that there were no statistical differences in energy expenditure for the same activity measured in the morning and the afternoon. Though we did not measure these sedentary activities into the evening, it is likely that these values would not be significantly different from the obtained values. Therefore, our repeated measurement design allowed us to demonstrate the stability of the energy expenditure of sedentary behaviors. This is important because it will allow researchers to have confidence in estimating the energy cost of sedentary behaviors across the day.

The thermic effect of food cannot be ignored when interpreting these analyses. The thermic effect of food reaches a plateau 20–30 minutes after a meal and elevates energy expenditure ∼15–30% for at least 3 hours and then gradually decreases back to baseline levels by about 5 hours. [42], [43] According to this model, the thermic effect of food should have had a similar effect on energy expenditure during the 0.5–2.5 hour postprandial measurement period. Therefore, the greatest rise in energy expenditure should have occurred just after the meal, which would be during each of the reclining activities in both the morning and afternoon sessions. Despite the fact that there was an ∼18%–30% increase in energy expenditure from the basal assessment to the sedentary activities that was sustained across the two hour assessment of sedentary behaviors, the energy cost of reclining was shown to be significantly lower than that of the other sitting behaviors (likely due to the decreased cost of being supine). Therefore, although the thermic effect of food likely contributed to the increase in energy expenditure, it does not totally account for the increase in energy expenditure measured after the meal. Thus, the energy expenditure values in this study are a combination of the thermic effect of food and energy required to engage in the sedentary behaviors. In this way, we provide accurate estimates of an individual’s energy expenditure in their usual state, sitting postprandially.

The examination of the postprandial energy expenditure during both the reclining and seated positions also provided an opportunity to discern how much of the increase in energy expenditure above BMR was due to sitting posture/activity per se and that due to thermic effect of food. The mean energy expenditure of the common seated activities was about 7% greater than reclining, or 0.07 kcal/kg/hr. This is an important calculation as it indicates that the average person expends only about 50 kcal/day (0.07 kcal/kg/hr x 9 hr x 79 kg) above resting (reclining) and the thermic effects of food, during an average day with 9 hours of sitting.

The findings demonstrate that the energy expended during typical sedentary behaviors is very narrowly bounded around ∼1.0 METs. It has been suggested that sedentary behavior be defined by activities expending 1.5 or less METs (Fig. 1). [23], [44] Data from the current study suggests that, on average, common sedentary behaviors expend between 0.97 and 1.06 METs. Therefore, the data from the current study supports the proposition that activity expending ≤1.5 METs can be safely defined as sedentary behavior. However, while 1.5 METs as a “sedentary threshold” may conservatively encapsulate all of the sedentary behaviors, it also may be too high and overlap with the lower threshold of energy expenditure defined as low-intensity physical activity (LIPA). LIPA are activities such as standing, walking, or stretching, and is generally regarded as activity expending more energy than sedentary behaviors, yet are below moderate intensity physical activity. Distinguishing between sedentary behavior and LIPA is important because engaging in LIPA may be a very specific recommendation for replacing sedentary time. [45], [46].

There are a number of strengths to the study. First, the most common forms of sedentary activities performed by adults were incorporated. Second, the study was conducted in a metabolic chamber. An advantage of the metabolic chamber method is that unlike the use of metabolic carts the volunteers are unencumbered and can move freely without wearing a device on the head and body, and energy expenditure can be measured comfortably for long epochs. Third, the study incorporated meals into the study design which presented the opportunity to assess the putative greater energy expenditure caused by sitting versus reclining in the most relevant nutritional conditions. Previous studies assessing the energy expenditure of activities have not incorporated this variable, decreasing the ecological utility of their studies. Fourth, long epochs were used. This allowed for fidgeting and other naturally occurring movements typical during sitting (e.g. weight shifts, scratching, limb movement to increase comfort, etc.) to occur. Given that sitting energy expenditure was close to 1.0 METS and within 7% of reclining, there was no evidence that these movements have a significant impact on the total energy used to sit. Fifth, the same activities were conducted in both the morning and afternoon, allowing for an assessment of the stability of energy estimates to be obtained. More importantly, this study design allowed for an “average sitting energy expenditure” to be calculated and available for use as a reference for the growing field that focuses on sedentary (sitting) behaviors.

There are several potential limitations to the study. The study could suffer from order effects because the order of the sedentary activities was not randomized. However, the data show that the values are very similar both between behaviors and between the morning and afternoon sessions. Therefore, it is unlikely that physiologically meaningful differences existed between the energetics of reading, typing, and watching TV. The study can also be thought to be limited in generalizability because it was conducted with an African American sample. In energy expenditure studies that have assessed the influence of ethnicity, there is some data to suggest that African Americans have a slightly lower resting metabolic rate compared to European Americans.[47]–[49] However, racial differences dissipate when accounting for organ sizes. [50] Data from the current study also suggest that concerns of limited generalizability are unwarranted. For example, similar values were found for BMR in the current study (0.86 kcal/kg/hr) compared to others (0.84–0.99 kcal/kg/hr).[25]–[27], [39], [41] Finally, we used MET units and energy expenditure per kg of total body weight to account for weight differences. The MET level of fasting or fed metabolic rate in the sedentary state (lying down or sitting) is likely dependent on BMI among other factors, and could shift the values up or down depending on body composition, which is beyond the scope of this paper.

Overall, the data from the current study provide energy expenditure values for common sedentary activities. The findings demonstrate that the energy expenditure for these activities does not differ across the day and that sedentary behaviors generally require minimally, but significantly, more energy compared to reclining. Participants were in the postprandial state for most of the activities and natural movements when sitting and reclining (e.g. fidgeting, leg shaking, shifting) were allowed to occur because the metabolic chamber was used. Therefore, the values of ∼1.0 METs for sitting and reclining likely represent accurate values for typical sitting energy expenditure. Most importantly, our results support the notion that common seated activities are almost always *well less than* 1.5 METs. Researchers can utilize this data to estimate the energy cost of these common sedentary behaviors. In summary, regardless of the sedentary activity or time of day, and taking into consideration the thermic effect of food, energy expenditure averaged 0.97 METs when reclined and ∼1.04 METs when seated. Therefore, we believe that our conclusion that sedentary behaviors are close to 1.0 METs is generalizable to similar populations, while we recognize the need for replication with more diverse samples to determine if more subtle differences exist.
