# Correction: The Energy Expenditure of Sedentary Behavior: A Whole Room Calorimeter Study

**DOI:** 10.1371/annotation/b66e5f0b-7898-4460-b088-d886a23fed29

**Published:** 2013-07-01

**Authors:** Robert L. Newton, Hongmei Han, Theodore Zderic, Marc Hamilton

The name of the fourth author was spelled incorrectly. The correct name is: Mark T. Hamilton. The correct citation is: Newton RL Jr, Han H, Zderic T, Hamilton MT (2013) The Energy Expenditure of Sedentary Behavior: A Whole Room Calorimeter Study. PLoS ONE 8(5): e63171. doi:10.1371/journal.pone.0063171. The correct abbreviation in Author Contributions is: MTH.

In addition, there was an error in Reference 38. The correct reference is: 38. Nguyen T, de Jonge L, Smith SR, Bray GA (2003) Chamber for indirect calorimetry with accurate measurement and time discrimination of metabolic plateaus of over 20 min. Med Biol Eng Comput 41: 572-578.

